# Temporal changes in mortality and risk factors of candidemia in intensive care unit patients

**DOI:** 10.1186/s12879-026-13759-9

**Published:** 2026-06-06

**Authors:** Burak Kizilcay, Zeynep Cizmeci, Gulsum Oya Hergunsel, Zafer Cukurova, Kadriye Kart Yasar, Zuhal Yesilbag

**Affiliations:** 1 Department of Infectious Diseases and Clinical Microbiology, Bakırköy Dr. Sadi Konuk Training and Research Hospital, University of Health Sciences, Istanbul, Turkey; 2 Department of Medical Microbiology, Bakırköy Dr. Sadi Konuk Training and Research Hospital, University of Health Sciences, Istanbul, Turkey; 3 Department of Anesthesiology and Reanimation, Bakırköy Dr. Sadi Konuk Training and Research Hospital, University of Health Sciences, Istanbul, Turkey

**Keywords:** Candidemia, Intensive care unit, Non-albicans *Candida*, Antifungal resistance, Mortality

## Abstract

**Background:**

*Candida* species are major causes of hospital-acquired bloodstream infections associated with high mortality in critically ill patients. This study aimed to evaluate temporal changes in species distribution, antifungal resistance, clinical outcomes, and mortality predictors in ICU patients with candidemia.

**Methods:**

This retrospective study included 250 ICU patients with candidemia between January 2018 and January 2022. The study period was divided into two cohorts (2018–2020, *n* = 80; 2020–2022, *n* = 170). Clinical, laboratory, and microbiological characteristics were compared. Multivariable analysis was performed to identify independent predictors of mortality.

**Results:**

The incidence density of candidemia significantly decreased from 4.32 to 3.10 per 1,000 ICU patient-days (IRR: 0.72; %95 CI: 0.55–0.94; *p* = 0,014). A shift toward non-albicans *Candida* species was observed, while antifungal resistance rates remained stable. Candida score values and empirical antifungal therapy rates were lower in the later period. Despite increased central venous catheter use, catheter-related candidemia decreased. Overall mortality was similar between periods; however, during the second period, mortality was higher in patients with SARS-CoV-2 infection than in those without infection [100/105 (95.2%) vs. 43/65 (66.2%), *p* < 0.001]. In multivariable analysis, SOFA score was independently associated with mortality in the early period (OR: 1.20, 95% CI: 1.01–1.43; *p* = 0.043), whereas SARS-CoV-2 infection (OR: 20.43, 95% CI: 3.53–118.14; *p* = 0.001), Candida score (OR: 0.19, 95% CI: 0.06–0.50; *p* = 0.003), lactate level at ICU admission (OR: 5.23, 95% CI: 1.55–17.57; *p* = 0.007), and urea level at ICU admission (OR: 1.01, 95% CI: 1.00–1.02; *p* = 0.030) were independent predictors in the later period.

**Conclusion:**

The high mortality observed among patients with SARS-CoV-2–associated candidemia underscores the need for heightened diagnostic awareness in deteriorating ICU patients. Early diagnostic work-up, timely initiation of antifungal therapy, and reassessment of treatment according to microbiological findings are important in this setting. Hospital-specific identification of preventable risk factors may also help optimize candidemia management and infection control strategies.

## Introduction

Candidemia is a serious and potentially life-threatening bloodstream infection, particularly among critically ill patients admitted to the intensive care unit (ICU) [1]. The true incidence of candidemia remains difficult to determine due to the limited sensitivity of blood cultures (50–70%), insufficient validation of PCR-based diagnostics, and the limited availability of adjunctive tests such as β-D-glucan [2]. In ICU surveillance data from the United States, *Candida* spp. have been reported to account for approximately 5–10% of all bloodstream infections [3, 4]. Studies from Turkey have similarly reported increasing incidence rates ranging from 1 to 5.4 cases per 10,000 patient-days [5, 6]. According to recent CDC population-based surveillance data from the United States, the overall incidence of candidemia during 2017–2021 was 7.4 cases per 100,000 population, and all-cause in-hospital mortality was 32.6% [7]. Similarly, the multinational European ECMM Candida III cohort reported an overall mortality rate of 40.4% among patients with candidemia [8]. Despite advances in antifungal therapy and critical care management, these data confirm that candidemia continues to carry a substantial mortality burden, particularly among critically ill ICU patients [4, 9]. A recent systematic review and meta-analysis of ICU patients identified broad-spectrum antibiotic exposure, blood transfusion, *Candida* colonization, central venous catheterization, total parenteral nutrition, and prolonged ICU stay as major factors associated with invasive *Candida* infection. Because these factors often coexist, they are better interpreted as interrelated host- and treatment-related risk clusters rather than isolated causal determinants [10].

The relationship between Severe Acute Respiratory Syndrome Coronavirus 2 (SARS-CoV-2) infection and invasive candidiasis remains incompletely understood [11]. However, critically ill patients with SARS-CoV-2 infection frequently develop secondary infections, partly related to disease severity and immunomodulatory therapies [12, 13]. Moreover, several factors associated with severe SARS-CoV-2 infection—including advanced age, malignancy, immunosuppression, and diabetes mellitus—are also well-recognized risk factors for candidemia [14]. Early recognition of candidemia and timely initiation of appropriate antifungal therapy in high-risk patients are crucial for reducing mortality in this population [15–17].

In this study, we aimed to evaluate temporal changes in the incidence of candidemia, the distribution of *Candida* species and antifungal resistance patterns, clinical factors associated with mortality, and the influence of concomitant SARS-CoV-2 infection on mortality among patients who developed candidemia while being treated in the intensive care unit during two consecutive study periods.

## Materials and methods

### Study design and exclusion criteria

This retrospective cross-sectional study was conducted in the adult intensive care units of a tertiary-care training and research hospital affiliated with the University of Health Sciences in Istanbul, Türkiye. The hospital serves as a referral center for a large urban population and receives patients from surrounding districts. ICU patients diagnosed with candidemia between January 2018 and January 2022 were included. The study period was divided into two intervals: January 2018–January 2020 and January 2020–January 2022. Patients younger than 18 years, those without candidemia, and those who developed candidemia within the first 48 h of ICU admission were excluded.

Candidemia was defined as the isolation of *Candida* spp. from at least one blood culture obtained ≥ 48 h after ICU admission. For patients with multiple candidemia episodes, only the first episode was included in the analysis, regardless of whether subsequent episodes were caused by the same or a different *Candida* species. Subsequent episodes were excluded to avoid duplicate patient-level observations. Demographic, clinical, laboratory, and microbiological data were retrospectively retrieved from electronic medical records. Data were retrospectively retrieved from electronic medical records and included demographic characteristics, underlying comorbidities, ICU-related exposures and invasive procedures, prior ICU or hospital exposure, antifungal treatment data, microbiological findings, SARS-CoV-2 infection status, severity scores, and laboratory parameters. Demographic characteristics and underlying comorbidities were recorded based on information available before candidemia onset. ICU-related exposures and invasive procedures, including central venous catheterization, endotracheal intubation, total parenteral nutrition, renal replacement therapy, and gastrointestinal surgery, were recorded if present before or at the time of candidemia onset. Laboratory parameters included leukocyte, neutrophil and lymphocyte counts, hemoglobin, platelet count, urea, creatinine, alanine aminotransferase, aspartate aminotransferase, C-reactive protein, procalcitonin, lactate, ferritin, and D-dimer levels, and were evaluated separately at ICU admission and at candidemia onset, when available. APACHE II and SOFA scores were obtained from daily ICU records in the electronic medical record system, and the values recorded on the day of candidemia onset were used for analysis. The Candida score was calculated at candidemia onset using available clinical and microbiological data. The colonization index was calculated using non-blood culture results available before or at the time of candidemia onset. Microbiological variables, including *Candida* species, isolation from non-blood samples, and antifungal susceptibility results, were recorded for the index candidemia episode. Antifungal treatment variables and mortality outcomes were assessed during follow-up after candidemia diagnosis.

### Definitions

The incidence of candidemia was calculated as the number of cases per 1,000 ICU patient-days. The Candida score was calculated based on total parenteral nutrition use, history of surgery, multifocal *Candida* colonization, and severe sepsis (1, 1, 1, and 2 points, respectively), as described by León et al. [18]. The colonization index was defined as the ratio of culture-positive sites to the total number of cultured sites as previously described by Pittet et al. [19]. *Candida* colonization was assessed retrospectively from clinically indicated non-sterile site cultures; routine surveillance cultures for *Candida* colonization were not performed in our ICU. Catheter-related candidemia was defined as candidemia occurring in a patient with a central venous catheter and no apparent alternative source of infection. When available, catheter involvement was supported by isolation of the same *Candida* species from the catheter tip or catheter-related cultures, or by a differential time to positivity of ≥ 2 h between catheter-drawn and peripheral blood cultures. Presumed infection foci were reviewed retrospectively by three infectious diseases specialists using clinical findings, microbiological results, catheter culture data, imaging findings, and electronic medical records, and final classification was made by consensus. However, *Candida* isolation from non-blood sites alone was not considered sufficient to define a definite candidemia source. Empirical antifungal therapy was defined as systemic antifungal treatment initiated before definitive identification of the causative microorganism, either after detection of yeast in blood culture or based on clinical signs and risk factors suggestive of invasive candidiasis.

Blood cultures were incubated using the BacT/Alert 3D automated system (bioMérieux, France). Identification of *Candida* isolates was performed using conventional culture media, the germ tube test, and the VITEK 2 Compact system (bioMérieux, Marcy-l’Étoile, France). Antifungal susceptibility testing, when performed, was conducted using the VITEK 2 Compact system and interpreted according to CLSI clinical breakpoints where applicable. CLSI M60 performance standards were used for interpretation when species-specific clinical breakpoints were available [20]. Isolates categorized as susceptible dose-dependent (SDD) were not classified as resistant in the resistance analyses. Susceptibility data were reported only for isolates with available and interpretable antifungal susceptibility results.

### Statistical analysis

Statistical analyses were performed using IBM SPSS Statistics version 25.0 (IBM Corp., Armonk, NY, USA). Data were summarized using descriptive statistics. Patients with missing data were not excluded from the entire study. Analyses were performed using available data for each variable, and no imputation was performed for missing values. Therefore, the number of observations may vary across analyses depending on the availability of data for each variable. Categorical variables were analysed using the chi-square or Fisher’s exact test. Continuous variables were assessed for normality using the Kolmogorov–Smirnov test and analysed using the Student’s t-test or Mann–Whitney U test, as appropriate. The statistical analyses were planned according to the main objectives of the study. Candidemia cases were first compared between the two study periods. Subsequently, among patients in the second study period, those with concomitant SARS-CoV-2 infection were compared with those without SARS-CoV-2 infection. Mortality-associated factors were evaluated using in-hospital mortality as the primary endpoint, and 28-day mortality after candidemia onset was assessed as an additional outcome. Variables identified in multivariable models were interpreted as independent predictors or mortality-associated factors rather than as causal determinants. Variables associated with mortality in univariate analyses were further evaluated using multivariable Cox regression to identify independent predictors. A *p* value < 0.05 was considered statistically significant.

### Ethics statement

The authors confirm that the ethical standards of the journal, as outlined in the author guidelines, were followed. This study was conducted in accordance with the principles of the Declaration of Helsinki and was approved by the Clinical Research Ethics Committee of the University of Health Sciences Türkiye, Bakırköy Dr. Sadi Konuk Training and Research Hospital (protocol code: 2022/20 − 11; approval date: 17 October 2022). As the study was retrospective in design, the requirement for informed consent was waived by the ethics committee.

## Results

A total of 250 patients with candidemia who were hospitalised in the ICU of our hospital between January 2018 and January 2022 were included in the study. Eighty patients were treated during the first study period (January 2018–January 2020), and 170 during the second study period (January 2020–January 2022). Among the 170 patients in the second study period, 105 (61.8%) had concomitant SARS-CoV-2 infection, while 65 (38.2%) were admitted to the ICU for other clinical conditions. SARS-CoV-2–positive patients were not admitted to the ICU solely on the basis of SARS-CoV-2 positivity. ICU admission during the pandemic period was based on the national COVID-19 management algorithm of the Turkish Ministry of Health and reflected clinical severity, including severe radiological pneumonia findings, respiratory failure or increasing oxygen requirement, clinical deterioration, hemodynamic instability, sepsis, or acute organ dysfunction.

### Comparison of candidemia cases between the two study periods

#### Temporal comparison of candidemia incidence, patient characteristics, treatment variables, and outcomes

In the first study period, candidemia developed in 80 of 4,200 ICU patients (1.9%), corresponding to an incidence density of 4.32 per 1,000 ICU patient-days. In the second period, 170 of 7,900 patients developed candidemia (2.15%), corresponding to an incidence density of 3.10 per 1,000 ICU patient-days. The reduction in incidence density between the two periods was statistically significant (incidence rate ratio [IRR]: 0.72; 95% CI: 0.55–0.94; *p* = 0.014). Demographic characteristics were similar between the two periods (Table 1). In the first period, the mean length of hospital stay prior to ICU admission was 21.16 ± 27.74 days, and the mean ICU stay was 16.86 ± 12.54 days. The mean time from ICU admission to candidemia was 9.41 ± 9.84 days in the first period and 11.81 ± 10.21 days in the second period. The mean length of hospital stay before ICU admission and ICU stay in the second period were 9.19 ± 10.72 and 18.79 ± 14.50 days, respectively. APACHE II scores were higher in the second period, whereas SOFA scores were similar between the periods. Endotracheal intubation and central venous catheter use were more frequent in the second period, while gastrointestinal surgery and total parenteral nutrition were more common in the first period. Among patients with a central venous catheter at candidemia onset, the median duration of central venous catheter placement before candidemia onset was 10 days (IQR, 5–12.75) in the first study period and 7 days (IQR, 4–11) in the second study period; however, this difference was not statistically significant (*p* = 0.090). Presumed catheter-related candidemia was identified in 13 patients (16.3%) in the first study period and 5 patients (2.9%) in the second study period (*p* < 0.001). *Candida spp.* isolation from non-blood sites, including urine, tracheal aspirate, and wound samples, was recorded for colonization assessment and reported separately as non-blood *Candida* isolation/colonization. These findings were not interpreted as evidence of invasive urinary tract candidiasis, pulmonary candidiasis, wound-related candidiasis, or presumed candidemia foci (Table 1).

**Table 1:** Comparison of candidemia cases between the two study periods

	First study period (*n* = 80)	Second study period (*n* = 170)	*p*
**Sex, ** ***n*** ** (%)**			
Male	42 (52.5)	94 (55.3)	0.679
Female	38 (47.5)	76 (44.7)	
**Age**,** years (mean ± SD)**	64.54 ± 17.98	64.72 ± 14.95	0.934
**Underlying comorbidities**,** n (%)**			
Diabetes mellitus	20 (25)	54 (31.8)	0.274
Hypertension	37 (46.3)	80 (47.1)	0.905
Heart failure	9 (11.3)	5 (2.9)	0.008*
Chronic Obstructive Pulmonary Disease (COPD)	13 (16.3)	15 (8.8)	0.082
Coronary artery disease	17 (21.3)	33 (19.4)	0.735
Chronic kidney disease (CKD)	12 (15)	29 (17.1)	0.682
Renal replacement therapy (RRT)	6 (7.5)	22 (13)	0.198
Cerebrovascular disease	13 (16.3)	24 (14.1)	0.658
Rheumatologic disease	4 (5)	13 (7.6)	0.438
Malignancy	23 (28.7)	41 (24.1)	0.434
**APACHE II**,** median (IQR)**	20.5 (17-27.75)	24 (18–30)	0.022*
**SOFA score**,** median (IQR)**	10 (6–12)	10 (8–12)	0.128
**Invasive procedures**,** n (%)**			
Presence of central venous catheter (CVC)	46 (57.5)	139 (81.8)	< 0.001 *
Total parenteral nutrition (TPN)	33 (41.3)	33 (19.4)	< 0.001 *
History of gastrointestinal surgery	31 (38.8)	22 (12.9)	< 0.001 *
Endotracheal intubation	76 (95)	170 (100)	0.003*
***Candida spp.***, **n (%)**			0.45
*Albicans*	36 (45)	68 (40)	
Non*-albicans*	44 (55)	102 (60)	
**Isolation of*Candida*spp. from other sites**,** n(%)**	43 (53.8)	87 (51.2)	0.704
Urine	24 (30)	69 (40.6)	0.106
Tracheal aspirate	11 (13.8)	28 (16.5)	0.58
Wound	3 (3.8)	2 (1.2)	0.175
**Catheter-related candidemia**	13 (16.3)	5 (2.9)	< 0.001 *
**Duration of catheterization (days)** median (IQR)	10 (5–12.75)	7(4–11)	0.090
**Colonization index**,** median (IQR)**	0.25 (0-0.5)	0.3 (0-0.5)	0.333
**Candida score**,** median (IQR)**	3 (2–3)	2 (1.7-3)	0.001*
**Antifungal therapy**			
Number of patients receiving antifungal therapy, n (%)	55 (68.8)	95 (55.9)	0.19
Empirical antifungal therapy, n (%)	39 (48.8)	55 (32.4)	0.013*
Antifungal agents, n (%)			
- Azoles	24 (43.6)	56 (58.9)	0.138
- Echinocandins	29 (52.7)	38 (40)	
- Amphotericin B	2 (3.6)	1 (1.1)	
Change of antifungal therapy, n (%)	20 (36.4)	20 (21.5)	0.05*
Antifungal therapy after modification, n (%)			
-Azoles	7 (35)	4 (20)	0.521
-Echinocandins	12 (60)	14 (70)	
-Amphotericin B	1 (5)	2 (10)	
Total treatment duration, median (IQR)	16 (7–22)	11.5 (5–15)	0.027*
**Azole resistance**,** n (%)**	17 (24.6)	25 (19.1)	0.359
**Echinocandin resistance**,** n (%)**	4 (5.8)	4 (3.1)	0.347
**Bacteremia at the time of candidemia**,** n (%)**	20 (25)	14 (21.5)	0.625
**Bacteremia prior to candidemia**,** n (%)**	26 (32.5)	36 (21.2)	0.053
**Antibiotic use prior to candidemia**,** n (%)**	76 (95)	170 (100)	0.003*
- Cephalosporin use, n (%)	24 (32)	52 (52.7)	0.826
- Piperacillin–tazobactam use, n (%)	21 (28)	98 (57.6)	< 0.001*
- Carbapenem use, n (%)	50 (65.8)	152 (89.4)	< 0.001*
- Colistin use, n (%)	16 (21.3)	48 (28.2)	0.257
- Glycopeptide use, n (%)	45 (60)	108 (63.5)	0.599
- Metronidazole use, n (%)	12 (16)	15 (8.8)	0.098
- Fluoroquinolone use, n (%)	16 (21.3)	26 (15.3)	0.248
- Linezolid use, n (%)	4 (5.3)	25 (14.7)	0.036*
**Duration of antibiotic use prior to candidemia**,** median (IQR)**	14.5 (7–24)	13 (9–20)	0.574
**Mortality**,** n (%)**	69 (86.3)	143 (84.1)	0.661

Abbreviations: APACHE II, Acute Physiology and Chronic Health Evaluation II; CI, confidence interval; CKD, chronic kidney disease; COPD, chronic obstructive pulmonary disease; CVC, central venous catheter; ICU, intensive care unit; IQR, interquartile range; OR, odds ratio; RRT, renal replacement therapy; SD, standard deviation; SOFA, Sequential Organ Failure Assessment; TPN, total parenteral nutrition; *Candida* spp., *Candida* species

#### Distribution of *Candida *species and antifungal resistance patterns across study periods

**Fig. 1:**
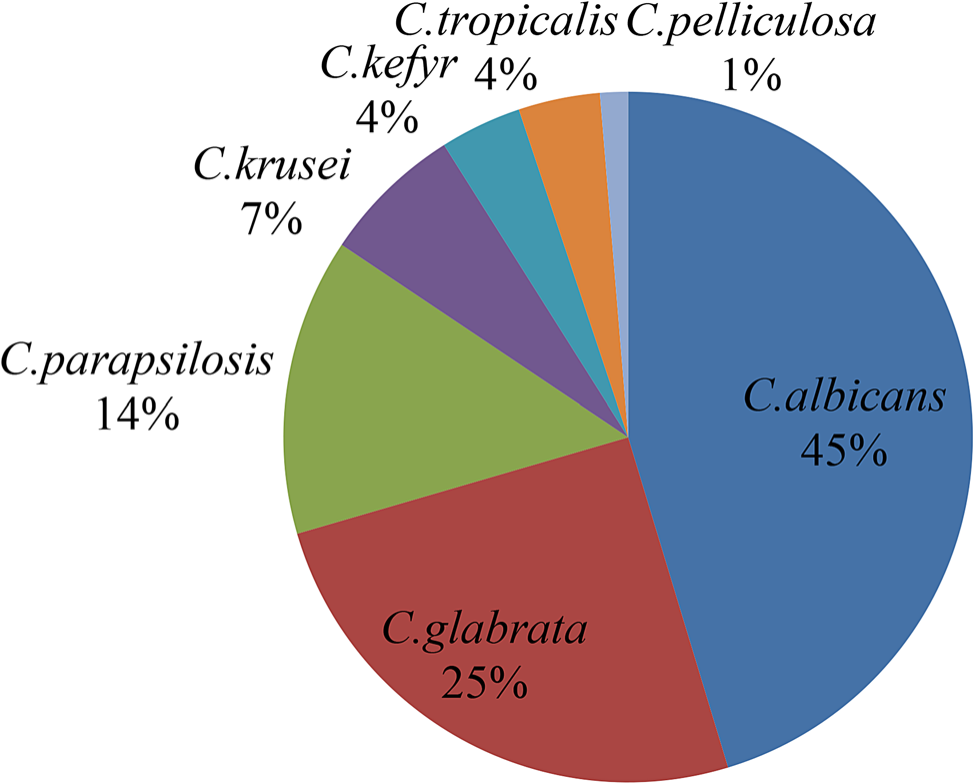
Distribution of *Candida* species in the first study period

**Fig. 2:**
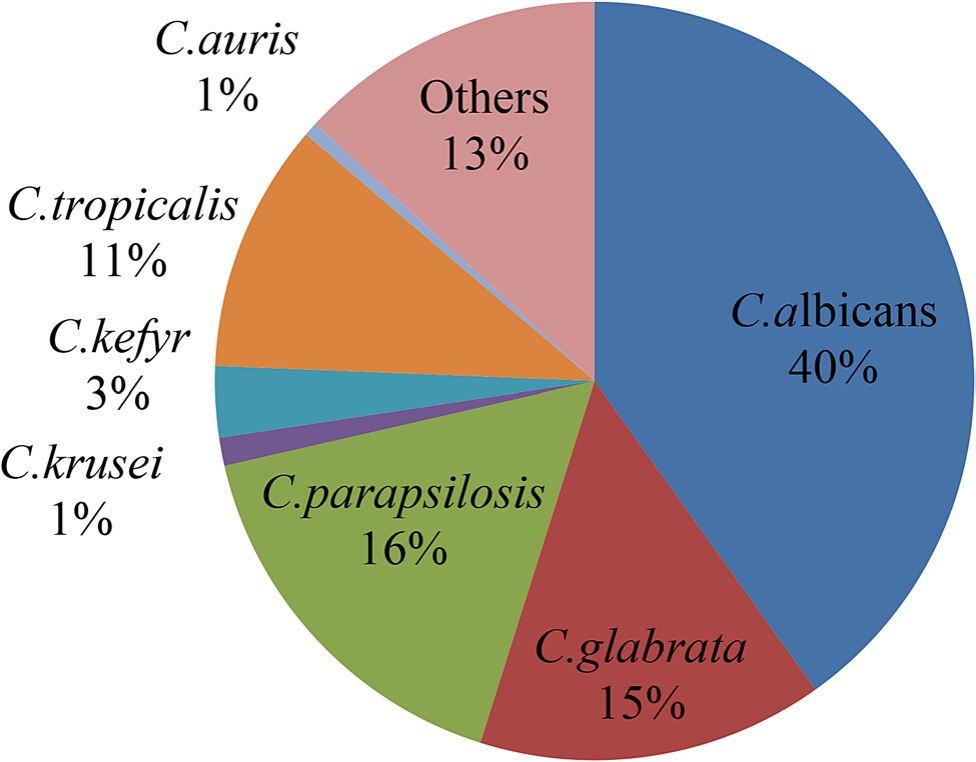
Distribution of *Candida* species during the second study period

The proportion of non-albicans *Candida* species was higher in the second period; however, this difference was not statistically significant (*p* = 0.45) (Table 1). Compared with the first period, the proportions of *C. glabrata* and *C. krusei* decreased, whereas *C. parapsilosis* and *C. tropicalis* increased (Figs. 1 and 2).

Species-specific antifungal resistance profiles according to the study periods are presented in Table 2. Among tested isolates, azole resistance in *C. glabrata* was significantly higher in the second study period than in the first study period [12/19 (63.2%) vs. 5/17 (29.4%), *p* = 0.043]. Azole resistance also differed among *C. tropicalis* isolates between the two periods [1/12 (8.3%) vs. 2/2 (100.0%), *p* = 0.033]. No significant period-related difference was observed in echinocandin resistance among the major *Candida* species.

**Table 2:** Species-specific azole and echinocandin resistance profiles of candidemia isolates according to study period

Species	First period azole resistance	Second period azole resistance	*p*	First period echinocandin resistance	Second period echinocandin resistance	*p*
Candida albicans	3/33 (9.1)	4/58 (6.9)	0.701	1/33 (3.0)	0/58 (0.0)	0.363
*Candida glabrata*	5/17 (29.4)	12/19 (63.2)	0.043*	3/17 (17.6)	2/19 (10.5)	0.650
*Candida parapsilosis*	2/8 (25.0)	3/26 (11.5)	0.570	0/8 (0.0)	2/26 (7.7)	1.000
*Candida tropicalis*	2/2 (100.0)	1/12 (8.3)	0.033*	0/2 (0.0)	0/12 (0.0)	1.000
*Candida kefyr*	0/3 (0.0)	0/3 (0.0)	1.000	0/3 (0.0)	0/3 (0.0)	1.000
*Candida krusei*	5/5 (100.0)	2/2 (100.0)	1.000	0/5 (0.0)	0/2 (0.0)	1.000

When *Candida* isolation from non-blood sites was evaluated, urine was the most common source in both periods. Catheter-related candidemia was significantly more frequent in the first period (*p* < 0.001) (Table 1). Antifungal susceptibility testing was performed for 69 of 80 isolates in the first period and for 131 of 170 isolates in the second period; no significant differences were observed between the periods in azole or echinocandin resistance.

#### Antifungal treatment practices, adjunctive treatment exposures, and pre-candidemia bacterial infections

Antifungal therapy was administered to 68.8% (*n* = 55) of patients in the first period and 55.9% in the second period, and empirical antifungal initiation was significantly more common in the first period (*p* = 0.01) (Table 1). Prior antibiotic use was observed in all patients in the second period and in 95% of those in the first period, most commonly carbapenems, glycopeptides, and piperacillin–tazobactam.

Immunomodulatory treatment exposure was also higher in the second study period. Corticosteroid use was significantly more frequent in the second period than in the first period [84/170 (49.4%) vs. 10/80 (12.8%), *p* < 0.001], and tocilizumab was used only during the second period [15/170 (8.8%) vs. 0/80 (0.0%), *p* = 0.006]. Bacteremia before candidemia was documented in 26/80 (32.5%) patients in the first study period and 36/170 (21.2%) patients in the second study period, yielding 27 and 37 bacterial isolates, respectively. Gram-negative bacteria accounted for 15/27 (55.6%) isolates in the first period and 22/37 (59.5%) in the second period. The most frequent isolates were *Enterococcus* spp. [5/27 (18.5%)], coagulase-negative staphylococci, *Klebsiella pneumoniae*, and *Acinetobacter baumannii* [each 4/27 (14.8%)] in the first period, and *Acinetobacter baumannii* [9/37 (24.3%)], *Enterococcus* spp. [8/37 (21.6%)], and *Pseudomonas aeruginosa* [5/37 (13.5%)] in the second period. Among Gram-negative isolates, carbapenem resistance was more frequent in the second period [20/22 (90.9%) vs. 6/15 (40.0%), *p* = 0.002].

### Comparison of candidemia cases with and without SARS-CoV-2 infection during the second study period

#### Clinical, microbiological, and bacteremia-related features according to SARS-CoV-2 status

Patients with SARS-CoV-2 infection were significantly older than those without infection (*p* = 0.019). No significant differences were observed between the groups in *Candida* species distribution, isolation from non-blood sources, colonization index, Candida score, or antifungal resistance (Table 3).

In the second study period, bacteremia before candidemia was documented in 16/65 (24.6%) patients without SARS-CoV-2 infection and 20/105 (19.0%) patients with SARS-CoV-2 infection (*p* = 0.441). The proportions of Gram-negative isolates [8/16 (50.0%) vs. 14/21 (66.7%), *p* = 0.336] and carbapenem resistance Gram-negative isolates [7/8 (87.5%) vs. 11/14 (78.6%), *p* = 1.000] did not differ significantly between the groups.

#### Antifungal treatment patterns and mortality according to SARS-CoV-2 status

Antifungal therapy was used more frequently in patients without SARS-CoV-2 infection (*p* = 0.015). Azoles were more commonly used in patients with SARS-CoV-2 infection, whereas echinocandins were more frequently used in those without infection (*p* = 0.003). Antifungal therapy modification was also more common in the non–SARS-CoV-2 group (*p* = 0.04). Mortality was significantly higher in patients with SARS-CoV-2 infection than in those without infection (*p* < 0.001) (Table 3).

**Table 3:** Comparison of candidemia patients with and without SARS-CoV-2 infection during the second study period

	Patients with candidemia and SARS-CoV-2 infection (*n* = 105)	Patients with candidemia without SARS-CoV-2 infection (*n* = 65)	*p*	OR	95% confidence interval
**Sex, ** ***n*** ** (%)**			0.737	1.113	0.59–2.07
Male	57 (56.9)	37 (54.3)			
Female	48 (43.1)	28 (45.7)			
Age, years (mean ± SD)	66.82 ± 14.06	61.32 ± 15.81	0.019*		
Underlying comorbidities, n (%)					
Diabetes mellitus	34 (32.4)	20 (30.8)	0.826	1.077	0.55–2.09
Hypertension	48 (45.7)	32 (49.2)	0.655	0.868	0.46–1.61
Heart failure	2 (1.9)	3 (4.6)	0.309	0.401	0.06–2.46
COPD	11 (10.5)	4 (6.2)	0.334	1.785	0.54–5.85
Coronary artery disease	20 (19)	13 (20)	0.879	0.941	0.43–2.05
CKD	19 (18.1)	10 (15.4)	0.648	1.215	0.52–2.80
RRT	18 (17.1)	6 (9.2)	0.150	2.034	0.76–5.42
Cerebrovascular disease	8 (7.6)	5 (7.7)	0.986	0.990	0.30–3.16
Rheumatologic disease	17 (16.2)	24 (36.9)	0.002*	0.330	0.16–0.68
*Candida spp.*, n (%)					
-*albicans*	37 (35.2)	31 (47.7)	0.107	1.676	0.89–3.14
*-*Non*-albicans*	68 (64.8)	34 (52.3)			
Isolation of *Candida* spp. from other sites, n(%)	53 (50.5)	34 (52.3)	0.816	0.929	0.50–1.72
Urine	42 (40)	27 (41.5)	0.843	0.938	0.50–1.76
Tracheal aspirate	18 (17.1)	10 (15.4)	0.764	1.138	0.49–2.64
Wound	0 (0)	2 (3.1)	0.071	0.375	0.30–0.45
Catheter-related candidemia	2 (1.9)	3 (4.6)	0.309	0.401	0.06–2.46
Colonization index, median (IQR)	0.50 (0-0.5)	0.33 (0-0.5)	0.950		
Candida score, median (IQR)	2 (2–3)	2 (1–3)	0.198		
Length of hospital stay prior to ICU admission (days), median (IQR)	6 (3–9)	8 (4-13.5)	0.032*		
Length of ICU stay (days), median (IQR)	16 (8.5–24)	14 (8-27.5)	0.601		
Time to candidemia (days), median (IQR)	10 (5-17.5)	6 (3-13.5)	0.021*		
Invasive procedures, n (%)					
Endotracheal intubation	105 (100)	65 (100)			
CVC	90 (85.7)	49 (75.4)	0.090	1.959	0.89–4.29
TPN	23 (21.9)	10 (15.4)	0.296	1.543	0.68–3.49
History of gastrointestinal surgery	6 (5.7)	16 (24.6)	**< 0.001**	0.186	0.06–0.50
Empirical antifungal therapy, n (%)	30 (28.6)	25 (38.5)	0.180	0.640	0.33–1.23
Antifungal therapy, n (%)	51 (48.6)	44 (67.7)	0.015*	0.451	0.23–0.86
Antifungal agents, n (%)					
- Azoles	38 (74.5)	18 (40.9)	0.003*		
- Echinocandins	13 (25.5)	25 (56.8)			
- Amphotericin B	0 (0)	1 (2.3)			
Change in antifungal therapy, n (%)	7 (13.7)	13 (31)	0.04*		
Antifungal therapy after modificitaion, n (%)					
- Azoles	2 (28.6)	2 (15.4)	0.478		
- Echinocandins	5 (71.4)	9 (69.2)			
- Amphotericin B	0 (0)	2 (15.4)			
Total duration of antifungal therapy (days), median (IQR)	7 (4–13)	14.5 (10-18.5)	0.001*		
Azole resistance, n (%)	11 (15.1)	14 (24.1)	0.189	0.558	0.23–1.34
Echinocandin resistance, n (%)	2 (2.7)	2 (3.4)	0.815	0.789	0.10–5.76
APACHE II, median (IQR)	25 (19-30.5)	22 (18-29.5)	0.268		
SOFA score, median (IQR)	11 (8–14)	9 (7–12)	0.036*		
Mortality, n (%)	100 (95.2)	43 (66.2)	**< 0.001**	10.23	3.63–28.79

Abbreviations: APACHE II, Acute Physiology and Chronic Health Evaluation II; CI, confidence interval; CKD, chronic kidney disease; COPD, chronic obstructive pulmonary disease; CVC, central venous catheter; ICU, intensive care unit; IQR, interquartile range; OR, odds ratio; RRT, renal replacement therapy; SD, standard deviation; SOFA, Sequential Organ Failure Assessment; TPN, total parenteral nutrition; *Candida* spp., *Candida* species

### Mortality analyses

#### Risk factors associated with mortality in the first study period

In the first study period, no significant differences were observed between survivors and non-survivors regarding age, sex, comorbidities, *Candida* species distribution, antifungal treatment, or resistance. However, APACHE II score, SOFA score, and lactate at admission, as well as procalcitonin, urea, creatinine, and lactate levels at candidemia onset, were significantly higher in non-survivors (*p* = 0.009, *p* = 0.006, *p* = 0.002, *p* = 0.029, *p* = 0.026, *p* = 0.018, and *p* = 0.038). In multivariable analysis, only SOFA score remained an independent predictor of in-hospital mortality ((OR: 1.20, 95% CI: 1.01–1.43; *p* = 0.043)).

### Risk factors associated with mortality among candidemic patients in the second study period

In the second study period, non-survivors were significantly older than survivors (*p* < 0.001), and SARS-CoV-2 infection was significantly more frequent among non-survivors (*p* < 0.001). No significant differences were observed between the groups regarding sex, comorbidities, *Candida* species distribution, isolation from non-blood samples, or antifungal resistance. However, antifungal therapy was less frequently administered in non-survivors than in survivors (52.4% vs. 74.1%, *p* = 0.038), and total antifungal treatment duration was significantly shorter among non-survivors (*p* < 0.001) (Table 4).

Urea and lactate levels at ICU admission, leukocyte count, procalcitonin, urea and lactate levels at candidemia onset, APACHE II score, SOFA score, and Candida score were significantly higher in non-survivors (*p* = 0.025, *p* < 0.001, *p* = 0.026, *p* = 0.001, *p* = 0.040, *p* < 0.001, *p* = 0.012, *p* < 0.001, and *p* = 0.004, respectively).

**Table 4:** Comparison of clinical, microbiological, treatment-related, and laboratory characteristics between survivors and non-survivors in the second study period

Variable	Non-survivors (*n* = 143)	Survivors (*n* = 27)	*p*
Sex: male/female, n (%)	78 (54.5) / 65 (45.5)	16 (59.3) / 11 (40.7)	0.651
Age, years, mean ± SD	66.69 ± 14.07	54.30 ± 15.43	< 0.001*
C. albicans/non-albicans, n (%)	58 (40.6) / 85 (59.4)	10 (37.0) / 17 (63.0)	0.732
Comorbidities: Diabetes mellitus, n (%)	49 (34.3)	5 (18.5)	0.107
Hypertension, n (%)	69 (48.3)	11 (40.7)	0.473
Coronary artery disease, n (%)	30 (21.0)	3 (11.1)	0.234
Chronic kidney disease, n (%)	27 (18.9)	2 (7.4)	0.146
Renal replacement therapy, n (%)	21/142 (14.8)	1/27 (3.7)	0.117
Cerebrovascular disease, n (%)	21 (14.7)	3 (11.1)	0.625
Malignancy, n (%)	31 (21.7)	10 (37.0)	0.087
Rheumatologic disease, n (%)	10 (7.0)	3 (11.1)	0.460
SARS-CoV-2 infection, n (%)	100 (69.9)	5 (18.5)	< 0.001*
*Candida* isolation from non-blood sites, n (%)	75 (52.4)	12 (44.4)	0.445
Urinary *Candida* isolation, n (%)	58 (40.6)	11 (40.7)	0.986
Tracheal aspirate *Candida* isolation, n (%)	24 (16.8)	4 (14.8)	0.800
Catheter-related candidemia, n (%)	5 (3.5)	0 (0.0)	1.000
Azole resistance, n/N (%)	22/104 (21.2)	3/27 (11.1)	0.237
Echinocandin resistance, n/N (%)	2/104 (1.9)	2/27 (7.4)	0.188
Candidemia preceded by bacteremia, n (%)	30 (21.0)	6 (22.2)	0.885
Hospitalization before ICU admission, n (%)	88 (61.5)	14 (51.9)	0.346
Central venous catheter, n (%)	118 (82.5)	21 (77.8)	0.559
Total parenteral nutrition, n (%)	28 (19.6)	5 (18.5)	0.898
History of gastrointestinal surgery, n (%)	16 (11.2)	6 (22.2)	0.117
Empirical antifungal therapy, n (%)	43 (30.1)	12 (44.4)	0.143
Any antifungal treatment, n (%)	75 (52.4)	20 (74.1)	0.038*
Antifungal therapy modification, n/N (%)	13/73 (17.8)	7/20 (35.0)	0.097
Leukocyte count at ICU admission, median (IQR)	12,790 (8090–18370)	11,320 (7290–15300)	0.246
Hemoglobin at ICU admission, median (IQR)	10.7 (8.8–12.5)	11.0 (8.9–12.6)	0.905
Neutrophil count at ICU admission, median (IQR)	11,330 (7190–15930)	9530 (5720–13400)	0.169
Lymphocyte count at ICU admission, median (IQR)	630 (400–1080)	770 (570–1200)	0.136
Platelet count at ICU admission, median (IQR)	222,000 (140000–315000)	284,000 (175000–403000)	0.105
Urea at ICU admission, median (IQR)	71 (44–132)	43 (21–121)	0.025*
Creatinine at ICU admission, median (IQR)	1.14 (0.73–2.32)	0.90 (0.69–1.92)	0.226
ALT at ICU admission, median (IQR)	23 (13–43)	28 (17–49)	0.277
AST at ICU admission, median (IQR)	39 (20–64)	36 (24–72)	0.815
CRP at ICU admission, median (IQR)	132 (70–217)	124 (68–155)	0.245
Procalcitonin at ICU admission, median (IQR)	0.73 (0.26–3.50)	0.36 (0.30–11.00)	0.566
Lactate at ICU admission, median (IQR)	1.9 (1.5–2.6)	1.3 (1.0–1.8)	< 0.001*
Ferritin at ICU admission, median (IQR)	713 (440.5–1333.8)	710 (350–1472)	0.551
D-dimer at ICU admission, median (IQR)	1.605 (0.900–3.380)	1.560 (0.703–5.348)	0.811
CRP at candidemia onset, median (IQR)	146.7 (91–237)	114 (50–190)	0.062
Leukocyte count at candidemia onset, median (IQR)	12,760 (8140–18530)	9030 (6100–13230)	0.026*
Hemoglobin at candidemia onset, median (IQR)	9.2 (8.1–10.4)	8.6 (8.0–9.9)	0.381
Lymphocyte count at candidemia onset, median (IQR)	850 (510–1340)	1020 (580–2190)	0.169
Neutrophil count at candidemia onset, median (IQR)	11,050 (6770–16660)	7590 (4000–10560)	0.004*
Ferritin at candidemia onset, median (IQR)	1093(540.75–2348.75)	728 (450–1536)	0.061
D-dimer at candidemia onset, median (IQR)	2.15 (1.315–3.90)	2.26 (1.49–7.10)	0.538
Platelet count at candidemia onset, median (IQR)	175,000(111000–255000)	203,000 (143000–336000)	0.141
ALT at candidemia onset, median (IQR)	33 (15–70)	33 (15–74)	0.890
AST at candidemia onset, median (IQR)	53 (29–106)	42 (21–95)	0.163
Procalcitonin at candidemia onset, median (IQR)	2.09 (0.69–7.22)	0.55 (0.14–3.115)	0.001*
Urea at candidemia onset, median (IQR)	82 (52–145)	44 (28–121)	0.040*
Creatinine at candidemia onset, median (IQR)	1.41 (0.82–2.82)	1.20 (0.49–2.15)	0.279
Lactate at candidemia onset, median (IQR)	2.7 (1.7–3.7)	1.6 (1.1–2.0)	< 0.001*
Colonization index, median (IQR)	0.50 (0–0.50)	0.00 (0–0.50)	0.267
Candida score, median (IQR)	2 (2–3)	2 (1–2)	0.004*
Duration of hospitalization before ICU admission, median (IQR)	6 (3–9.75)	12 (5.75–16.75)	0.006*
Length of ICU stay, median (IQR)	15 (8–24)	12 (7–29)	0.814
Day of candidemia onset, median (IQR)	9 (4–15)	7 (3–23)	0.969
Blood culture time to positivity/growth, hours, median (IQR)	96 (70–140)	90 (67–136)	0.720
Time from growth to treatment, hours, median (IQR)	0 (0–4)	0 (0–9.75)	0.836
First antifungal treatment duration, median (IQR)	7 (4–12)	15 (12–18.25)	< 0.001*
Second antifungal treatment duration, median (IQR)	7 (5.5–21)	14 (9–20)	0.152
Total antifungal treatment duration, median (IQR)	8.5 (5–13)	15.5 (15–27.75)	< 0.001*
APACHE II score, median (IQR)	25 (19–32)	20 (15–25)	0.012*
SOFA score, median (IQR)	11 (8–14)	8 (6–10)	< 0.001*

Multivariable analysis identified concomitant SARS-CoV-2 infection (*p* = 0.001, OR: 20.43, 95% CI: 3.53–118.14), higher Candida score (*p* = 0.003, OR: 0.19, 95% CI: 0.06–0.50), and higher urea (*p* = 0.03, OR: 1.01, 95% CI: 1.00–1.02) and lactate levels at ICU admission (*p* = 0.007, OR: 5.23, 95% CI: 1.55–17.57) as independent predictors of in-hospital mortality.

#### Risk factors associated with 28-day mortality after candidemia onset

In the multivariable analysis of risk factors affecting 28-day mortality in the first study period, day of candidemia onset, procalcitonin level at candidemia onset, lactate level at ICU admission, age, and isolation of *Candida* spp. from urine were identified as independent risk factors (*p* < 0.001, *p* = 0.001, *p* = 0.010, *p* = 0.008, and *p* = 0.001, respectively). In the multivariable analysis of risk factors affecting 28-day mortality in the second study period, age and antifungal therapy modification were identified as independent risk factors (*p* = 0.027 and *p* = 0.011, respectively) (Table 5).

**Table 5:** Multivariable analysis of risk factors associated with 28-day mortality after candidemia onset according to study period

Study period	Variable	OR	95% confidence interval	*p*
First period	Procalcitonin level at candidemia onset	1.027	1.010–1.043	0.001*
	Lactate level at ICU admission	1.114	1.026–1.210	0.010*
	Age	1.024	1.006–1.041	0.008*
	Day of candidemia onset	0.897	0.854–0.941	< 0.001*
	Urinary *Candida* isolation	3.311	1.585–6.917	0.001*
Second period	Age	1.022	1.002–1.042	0.027*
	Antifungal therapy modification	0.158	0.038–0.656	0.011*

## Discussion

In our study, the incidence density of candidemia significantly decreased from 4.32 to 3.10 per 1,000 ICU patient-days between the two study periods. Although several studies from Turkey and other countries have reported increasing candidemia incidence during the same timeframe, our findings demonstrated a decline in the later period [17, 21, 22].

During the second study period, ICU capacity and patient flow changed substantially with the incorporation of an additional pandemic hospital into our hospital system. The ICU rooms in this pandemic hospital were mainly single-occupancy, and infection control practices were intensified during this period. Although the crude patient-based proportion of candidemia was slightly higher in the second period, the incidence density calculated per 1,000 ICU patient-days was significantly lower. This apparent discrepancy may be explained by the fact that these two measures are calculated differently: the former reflects the number of candidemia cases among ICU admissions, whereas the latter also accounts for the total duration of ICU exposure. Therefore, the slight increase in patient-based proportion may reflect changes in ICU admissions and case-mix, particularly the increased number of critically ill patients with SARS-CoV-2 infection, while the lower patient-day–based incidence density may be related to expanded ICU capacity, single-occupancy ICU rooms, and strengthened infection control practices. Reduced surgical activity and fewer admissions of immunosuppressed patients may also have resulted in fewer individuals with classical candidemia risk factors. Demographic characteristics of candidemia cases were similar between the two study periods, consistent with findings reported by Cruz et al. [23].

The proportion of non-albicans *Candida* species among candidemia cases has increased over time [2, 24, 25]. A previous study from our institution reported an increase from 30% to 50% between 2013 and 2017 [26]. In our study, non-albicans *Candida* species were more frequent in the second period (60%) than in the first (55%), consistent with both the literature and our previous findings. This modest increase was mainly driven by higher proportions of *C. parapsilosis* and *C. tropicalis*, whereas *C. glabrata* and *C. krusei* decreased in the later period. High exposure to broad-spectrum antibiotics, extensive use of invasive devices, and changes in ICU case-mix during the pandemic may have contributed to this pattern, as these factors are well-recognized contributors to invasive candidiasis in critically ill patients [10, 27, 28]. In particular, the increase in *C. parapsilosis* may be clinically relevant, given its association with catheter-related infection, biofilm formation, total parenteral nutrition, and healthcare-associated transmission [29]. However, because the overall albicans/non-*albicans Candida* difference was not statistically significant, this finding should be interpreted as a descriptive temporal trend rather than a definitive epidemiological shift.

In our study, CVC use was higher in the second study period, whereas gastrointestinal surgery and total parenteral nutrition were more frequent in the first period. This likely reflects the predominance of surgical patients in the earlier period and the reduced number of elective procedures during the later period. Despite higher CVC utilization, catheter-related candidemia was less frequent in the second period. Among patients with a CVC at candidemia onset, the median duration of CVC placement before candidemia onset was numerically shorter in the second period than in the first period; however, this difference did not reach statistical significance. Therefore, shorter catheter dwell time may have contributed to the lower frequency of catheter-related candidemia in the second period, but this association could not be statistically demonstrated in our cohort. In addition, these infection control measures may have particularly affected catheter-related events by reducing catheter colonization.

No differences were observed in antifungal treatment choice between the two study periods. However, empirical antifungal therapy was initiated less frequently in the later period. This may partly reflect the limited early awareness during the pandemic that SARS-CoV-2 infection and its ICU management could increase the risk of candidemia. In addition, classical candidemia risk factors changed during the second study period; gastrointestinal surgery and total parenteral nutrition were less frequent than in the first period, and Candida scores were lower. These changes may have reduced the perceived pre-test probability of candidemia and contributed to lower empirical antifungal initiation. Within the second study period, antifungal therapy was administered more frequently in patients without SARS-CoV-2 infection than in those with SARS-CoV-2 infection, which may reflect differences in clinical suspicion, timing of diagnosis, and underlying ICU case-mix. The higher echinocandin use in patients without SARS-CoV-2 infection may reflect a more typical healthcare-associated candidemia risk profile and greater initial clinical suspicion for invasive candidiasis in this group. Antifungal resistance rates, including fluconazole resistance, were similar between the periods, consistent with the findings of Routsi et al. [21]. However, species-level analysis showed a significant increase in azole resistance among *C. glabrata* isolates during the second study period; this finding should be interpreted cautiously because susceptibility testing was not available for all isolates and the number of tested isolates was limited for some species. In the first study period, the relatively high proportion of *C. glabrata* among candidemia episodes should also be considered when interpreting empirical azole use. Given the reduced azole susceptibility commonly associated with *C. glabrata* and the azole resistance observed in our isolates, empirical azole therapy may have carried a risk of suboptimal initial coverage in some patients. However, because of the retrospective design, this finding should be interpreted cautiously and cannot establish a direct relationship between empirical azole use, inadequate treatment, and mortality.

When antibiotic use was evaluated, all patients in the second study period received antibiotic therapy. Although the frequency of bacteremia before or during candidemia did not differ between periods, antibiotic use was significantly higher in the second period. One explanation may be that SARS-CoV-2–associated cytokine syndrome can mimic bacterial sepsis, complicating antimicrobial stewardship and clinical decision-making. Accordingly, previous studies have reported that more than 90% of patients with SARS-CoV-2 infection receive empirical antibiotic therapy [30]. Piperacillin–tazobactam, carbapenems, and linezolid were used more frequently in the second period, likely due to concern for secondary pulmonary infections. Increased workload during this period may also have reduced antimicrobial stewardship oversight, leading to more uncontrolled antibiotic use in the ICU.

Invasive candidiasis developing in critically ill patients with SARS-CoV-2 infection appears to reflect the combined effects of classical ICU-related risk factors and COVID-19–specific treatment and management practices. Although candidemia is the most frequently reported clinical form in studies of COVID-19–associated invasive candidiasis, catheter-related, urinary tract, and intra-abdominal involvement may also occur. Previous studies have suggested that severe COVID-19, prolonged ICU stay, mechanical ventilation, CVC use, broad-spectrum antibiotic exposure, renal replacement therapy, TPN, corticosteroid exposure, and immunomodulatory treatments may increase susceptibility to invasive candidiasis [27, 28]. A recent systematic review also reported that mortality among ICU patients with COVID-19–associated candidemia reached 68.4%, supporting the association between candidemia and severe critical illness in this population [28]. In a recent case–control study, antibiotic exposure and disease severity were identified as independent risk factors for healthcare-associated candidemia in adults hospitalized with SARS-CoV-2 infection, whereas corticosteroid and interleukin-6 inhibitor use were not independently associated with candidemia [38]. These observations are partly consistent with our findings. Patients with SARS-CoV-2–associated candidemia had higher SOFA scores, later candidemia onset, lower rates of antifungal treatment, shorter treatment duration, and markedly higher mortality than those without SARS-CoV-2 infection. However, not all classical candidemia risk factors were more frequent in this group; for example, gastrointestinal surgery was less common, and ICU stay, CVC use, TPN, and renal replacement therapy did not differ significantly between groups [27, 28, 31].

In-hospital mortality rates were high in both the first and second study periods, with no significant difference between them. Similar findings have been reported by Mastrangelo et al. and Routsi et al. [15, 21].

Within the second study period, patients with SARS-CoV-2–associated candidemia were significantly older than those without SARS-CoV-2 infection. This may reflect national management policies prioritizing hospitalisation of patients aged ≥ 65 years and the higher likelihood of ICU admission among elderly patients with comorbidities. Similar trends have been reported by Seagle et al., Karakök et al., Kayaaslan et al., and El Zakhem et al., although the differences were not statistically significant in those studies [17, 32–34]. Malignancy was more frequent in the non–SARS-CoV-2 group, whereas other comorbidities were similar between groups, consistent with previous reports [32–34].

In our study, candidemia developed later in patients with SARS-CoV-2 infection during the second study period than in those without infection. The median time to candidemia was 10 days in patients with SARS-CoV-2 infection and 6 days in those without infection. This later onset may be explained by the clinical course and cumulative ICU exposures of patients with severe SARS-CoV-2 infection. In our cohort, patients with SARS-CoV-2 infection had a shorter hospital stay before ICU admission but tended to remain in the ICU for a longer period, allowing ICU-related risk factors to accumulate over time. In addition, immunomodulatory treatments such as high-dose corticosteroids and tocilizumab were generally administered during the ICU course, and their potential effects on susceptibility to secondary fungal infections may have become more evident in the later phase of critical illness. Therefore, the later onset of candidemia in patients with SARS-CoV-2 infection may reflect the cumulative effect of prolonged ICU exposure, invasive procedures, broad-spectrum antibiotics, and immunomodulatory treatments rather than risk factors present at ICU admission. Although the rate of empirical antifungal initiation did not differ significantly between the groups, overall antifungal treatment was more frequent among patients without SARS-CoV-2 infection. Only 48% of patients with SARS-CoV-2–associated candidemia received antifungal therapy, compared with 67% of patients without infection.

Given that candidemia developed later in patients with SARS-CoV-2 infection, some patients may have died before culture results became available and antifungal therapy could be initiated, which may explain the lower treatment rates observed in this group. In our review of untreated cases, no separate subgroup was identified in which candidemia was recognized from blood culture results and antifungal therapy was withheld for an alternative predefined clinical reason. Antifungal therapy duration was also significantly shorter in patients with SARS-CoV-2–associated candidemia, with a median duration of 7 days compared with 14 days in patients without infection, further suggesting that some patients died before receiving treatment or shortly after therapy initiation. Regarding antifungal choice, azoles were used more frequently in patients with SARS-CoV-2 infection, whereas echinocandins were preferred in patients without infection. Modification of antifungal therapy based on culture results or clinical response was also more common in the non–SARS-CoV-2 group. Mortality was significantly higher among patients with SARS-CoV-2–associated candidemia. Similar findings were reported by Karakök et al.; however, Papadimitriou et al. did not observe a significant difference in mortality between the groups, which they attributed to the predominance of catheter-related candidemia and early source control through central venous catheter removal within 48 h [34, 35].

When risk factors affecting in-hospital mortality were evaluated, the SOFA score was identified as the only independent predictor of mortality in the first study period, whereas SARS-CoV-2 infection was found to be one of the independent risk factors in the second study period. Similarly, in the study by Kayaaslan et al., SARS-CoV-2 infection was also identified as an independent risk factor for mortality [17].

In the additional analysis of 28-day mortality after candidemia onset, advanced age was independently associated with mortality in both study periods, while procalcitonin (PCT) level at candidemia onset and lactate level at ICU admission were independent predictors in the first period. This association between advanced age and mortality is consistent with recent candidemia studies [36, 37]. The prognostic relevance of PCT and lactate is also supported by recent data showing that PCT may predict short-term mortality risk in candidemia and that lactate contributes to candidemia-specific 28-day mortality prediction [38, 39] These findings suggest that early mortality in ICU patients with candidemia is closely related to host vulnerability, systemic inflammatory burden, and hypoperfusion-related physiological derangement.

Day of candidemia onset and urinary isolation of *Candida* spp. were also associated with 28-day mortality in the first period; however, these variables should be interpreted cautiously, as they may reflect disease severity, timing of clinical deterioration, colonization burden, or urinary catheter exposure rather than direct causal effects. In the second study period, antifungal therapy modification was associated with lower 28-day mortality, supporting the potential importance of reassessing antifungal treatment after microbiological confirmation and according to clinical response. This finding is consistent with recent ICU candidemia studies emphasizing appropriate antifungal therapy, catheter removal, and source-control strategies as key determinants of short-term survival [40, 41].

## Limitations

This study has several limitations. First, its retrospective and single-centre design may limit the generalizability of the findings and may have resulted in incomplete clinical or laboratory data, potentially leading to information bias. Second, antifungal susceptibility testing was not performed for all Candida bloodstream isolates. This may be related to changes in laboratory practice during the study period, increased laboratory workload during part of the COVID-19 pandemic, and the fact that in some cases Candida growth was detected after the patient had died, limiting the clinical utility and prioritization of susceptibility testing. Therefore, resistance patterns should be interpreted cautiously and should not be considered definitive estimates. Third, presumed candidemia foci other than catheter-related candidemia could not be reliably assigned in all patients. In addition, changes in ICU admission policies, infection control practices, and clinical management strategies during the period characterized by widespread SARS-CoV-2 infection may have influenced the observed differences between study periods.

## Conclusions

In this ICU cohort, candidemia incidence density decreased during the second study period, although the clinical course of candidemia differed in several important aspects. Catheter-related candidemia decreased despite increased central venous catheter use, and a modest shift toward non-*albicans Candida* species was observed without a significant increase in overall antifungal resistance. Within the second study period, SARS-CoV-2–associated candidemia represented a distinct high-risk subgroup, characterized by later onset, lower antifungal treatment rates, shorter treatment duration, and markedly higher mortality. These findings suggest that, in critically ill patients with SARS-CoV-2 infection, candidemia may be difficult to recognize using conventional risk-based approaches alone, particularly when classical risk factors are less prominent and clinical deterioration is mainly attributed to severe COVID-19. Therefore, rather than attributing changes in resistance patterns directly to the pandemic, our findings support continuous ICU-based monitoring of *Candida* species distribution, antifungal susceptibility, timing of candidemia onset, and antifungal treatment practices, with particular attention to evolving risk profiles in critically ill patients with SARS-CoV-2 infection.

## Data Availability

The datasets used and/or analyzed during the current study are available from the corresponding author on reasonable request.

## Declarations

### Use of artificial intelligence

The authors declare that no artificial intelligence tools were used in the preparation of this manuscript.
